# Vaginal microbiota of American Indian women and associations with measures of psychosocial stress

**DOI:** 10.1371/journal.pone.0260813

**Published:** 2021-12-10

**Authors:** Joanna-Lynn C. Borgogna, Michael Anastario, Paula Firemoon, Elizabeth Rink, Adriann Ricker, Jacques Ravel, Rebecca M. Brotman, Carl J. Yeoman

**Affiliations:** 1 Department of Microbiology and Immunology, Montana State University, Bozeman, Montana, United States of America; 2 Department of Animal and Range Sciences, Montana State University, Bozeman, Montana, United States of America; 3 Department of Health Promotion and Disease Prevention, Florida International University, Miami, Florida, United States of America; 4 Fort Peck Community College, Poplar, Montana, United States of America; 5 Department of Health and Human Development, Montana State University, Bozeman, Montana, United States of America; 6 School of Public Health–Center for American Indian Health and School of Nursing, John Hopkins University, Baltimore, Maryland, United States of America; 7 Institute for Genome Sciences, University of Maryland School of Medicine, Baltimore, Maryland, United States of America; 8 Department of Microbiology and Immunology, University of Maryland School of Medicine, Baltimore, Maryland, United States of America; 9 Department of Epidemiology and Public Health, University of Maryland School of Medicine, Baltimore, Maryland, United States of America; University of Westminster, UNITED KINGDOM

## Abstract

Molecular-bacterial vaginosis (BV) is characterized by low levels of vaginal *Lactobacillus* species and is associated with higher risk of sexually transmitted infections (STI). Perceived psychosocial stress is associated with increased severity and persistence of infections, including STIs. American Indians have the highest rates of stress and high rates of STIs. The prevalence of molecular-BV among American Indian women is unknown. We sought to evaluate measures of psychosocial stress, such as historic loss (a multigenerational factor involving slavery, forced removal from one’s land, legally ratified race-based segregation, and contemporary discrimination) and their association with the vaginal microbiota and specific metabolites associated with BV, in 70 Northwestern Plains American Indian women. Demographics, perceived psychosocial stressors, sexual practices, and known BV risk factors were assessed using a modified version of the American Indian Service Utilization, Psychiatric Epidemiology, Risk and Protective Factors Project survey. Self-collected mid-vaginal swabs were profiled for bacterial composition by 16S rRNA gene amplicon sequencing and metabolites quantified by targeted liquid-chromatography mass spectrometry. Sixty-six percent of the participants were classified as having molecular-BV, with the rest being either dominated by *L*. *crispatus* (10%) or *L*. *iners* (24%). High levels of lifetime trauma were associated with higher odds of having molecular-BV (adjusted Odds Ratio (aOR): 2.5, 95% Credible Interval (CrI): 1.1–5.3). Measures of psychosocial stress, including historic loss and historic loss associated symptoms, were significantly associated with lifestyle and behavioral practices. Higher scores of lifetime trauma were associated with increased concentrations of spermine (aFC: 3.3, 95% CrI: 1.2–9.2). Historic loss associated symptoms and biogenic amines were the major correlates of molecular-BV. Historical loss associated symptoms and lifetime trauma are potentially important underlying factors associated with BV.

## Introduction

Racial disparities in the burden of sexually transmitted infections (STIs) continue to persist at unacceptable levels in the US [1–4]. American Indian (AI) women are disproportionately affected, having rates of *Neisseria gonorrhoeae* (GC), *Chlamydia trachomatis* (CT), *Treponema pallidum* (the causative agent of syphilis), *Trichomonas vaginalis* (TV) and HIV infection 1.5–6 times higher than non-Hispanic white women [1–4]. Racial misidentification, insufficient levels of surveillance, and under-reporting of urogenital diseases affect AI populations, and it is estimated that the prevalence of these STIs are at least 30–57% higher than documented [5–10]. Population-based surveys clearly indicate that these inequities cannot be attributed to behavioral practices, including sexual activity, age of first intercourse, condom use, and drug use [11–13], and the mechanisms affecting racial disparities in susceptibilities to female reproductive tract infections remain unclear.

We hypothesized that the vulnerability of AI women to high rates of STIs may be partially mediated by psychosocial stress [14]. Psychosocial stress occurs when an individual perceives an event as taxing, threatening, or otherwise harmful, and their coping resources as inadequate [15–18]. Thus, a stressful experience is individualized, and experiencing an event may be perceived as stressful for some but not others [15, 17, 19]. Stressors can take on many forms, including short-term demands such as occupational burdens and deadlines [15, 17–19] and chronic demands such as those that persist over an extended duration of time (e.g., caring for a partner with dementia), traumatic life events (e.g., experiencing a sexual assault, early childhood trauma, or death of a loved one), and demands derived from poverty or perceived discrimination [15, 17–20].

AIs have the highest rate of stress exposure in the US [19, 21–25], and this has been tied to historical loss (HLS) associated trauma, a multigenerational factor involving slavery, forced removal from one’s land, legally ratified race-based segregation, and contemporary discrimination [26–30]. Factors that may exacerbate the high rates of stress include the lowest national employment rates [31, 32], highest rates of poverty [33, 34], exposure to violent acts and repeated loss [14, 33], with death rates being as much as 46% higher than that of non-Hispanic whites [35]. AI women are particularly imperiled, reporting the most extreme rates of interpersonal (emotional, physical, or sexual abuse, and emotional, or physical neglect) trauma within the US [14, 26, 36–39], with over 84% reporting having had experienced some form of violence during their lifetime [40, 41].

Exposure to psychosocial stress, including community-level stressors, can elicit physiological responses leading to suppressed immune function, and increased susceptibility, severity, and persistence of infections, including STIs [16, 22, 42–51]. Psychosocial stress has also been linked to bacterial vaginosis (BV) [22, 45–47, 52], the most common gynecological morbidity in the US [53]. Molecular features of BV include a paucity of protective lactobacilli [54–57], an abundance of diverse anaerobes [58–61], a high vaginal pH (>4.5) [55], and high concentrations of metabolites called biogenic amines (BAs) [62–65]. The low-lactobacilli state of BV (recently termed molecular-BV [58]) is associated with adverse gynecological outcomes, including an elevated risk of acquisition of HIV [57, 58, 66, 67] and other STIs [68–71]. Conversely, vaginal microbiota dominated by protective lactobacilli are typically associated with positive health outcomes [62, 72–75].

Most BAs commonly associated with BV are microbially produced via specific amino acid decarboxylation reactions, a common acid- and oxidative-stress resistance mechanism [76]. BAs negatively affect growth and lactic acid production of lactobacilli [77], increase odds of BV [77], are associated with smoking [78], CT [79], and HPV [80], and improve the growth and virulence of several pathogens, including GC [81–84]. Further, physiological and emotional stress are known to elicit increases in levels of BAs in some tissues, including the brain and liver—a phenomenon described as the polyamine-stress response [85–88]. To date, it has not been examined if stress can affect BA concentrations in the vagina.

We hypothesized that high psychosocial stress may be associated with both vaginal microbiota and vaginal BAs, creating a vaginal environment susceptible to STIs. While the role of the vaginal microbiota in sexual and reproductive health is widely established, we are unaware of any study describing the vaginal microbiota of AI women. Therefore, in this study we utilized 16S rRNA gene amplicon sequencing to characterize the vaginal microbiota of AI women in a small cross-sectional cohort. We also performed targeted metabolomics to quantify the vaginal BAs and their amino acid precursors, and evaluated their relationship to HLS trauma, psychosocial stress, and behavioral practices of AI women.

## Results

### Participant characteristics and demographics

Participant characteristics and demographics are reported in Table 1. This study did not assess gender identity. The average age of participants was 30 years (SD: 7 years, range: 18–45 years). All participants reported being enrolled in a federally recognized Northern Plains Tribe, with four participants (5.7%) identifying as additionally having associate tribal membership. Most participants reported having had some college education (50%) compared to those that reported either having some or no high school (31%) or having received a high-school diploma or GED (19%) (p-values estimated using pairwise two-sample proportion z-test<0.04). Most participants identified homemaker as their sole (40%) or primary occupation (13%), self-reported as recipients of public assistance (63%) and indicated that they cohabitated with either a relative (40%) or their partner (30%).

**Table 1:** Participant demographics.

Participant Details	Total Participants N = 70 (%)
**Federal Tribe Recognition Status**	
Member	66 (94.3)
Member and Associate Member	4 (5.7)
**Age**	
18–28	32 (45.7)
29–39	28 (40)
40–45	10 (14.3)
**Highest education level**	
Some or no High School (no diploma/GED)	22 (31.4)
High School, 9–12 (diploma/GED)	13 (18.6)
College, 1–4 years (no degree)	25 (35.7)
College, 1–4 years (Associates or Bachelors)	8 (11.4)
Currently a college student	2 (2.9)
**On Public Assistance**	
On Public Assistance	44 (62.8)
**Primary Occupation**	
Employed/Self-employed	16 (22.9)
Homemaker	28 (40)
Homemaker, multiple occupations	9 (12.9)
Unemployed	15 (21.4)
No Answer	2 (2.9)
**Current Housing Situation**	
Lives with relative	28 (40)
Lives with partner	21 (30)
Lives with partner’s relative	7 (10)
Prefer not to answer	14 (20)

### Participants were predominately characterized by molecular-BV

**Fig 1:**
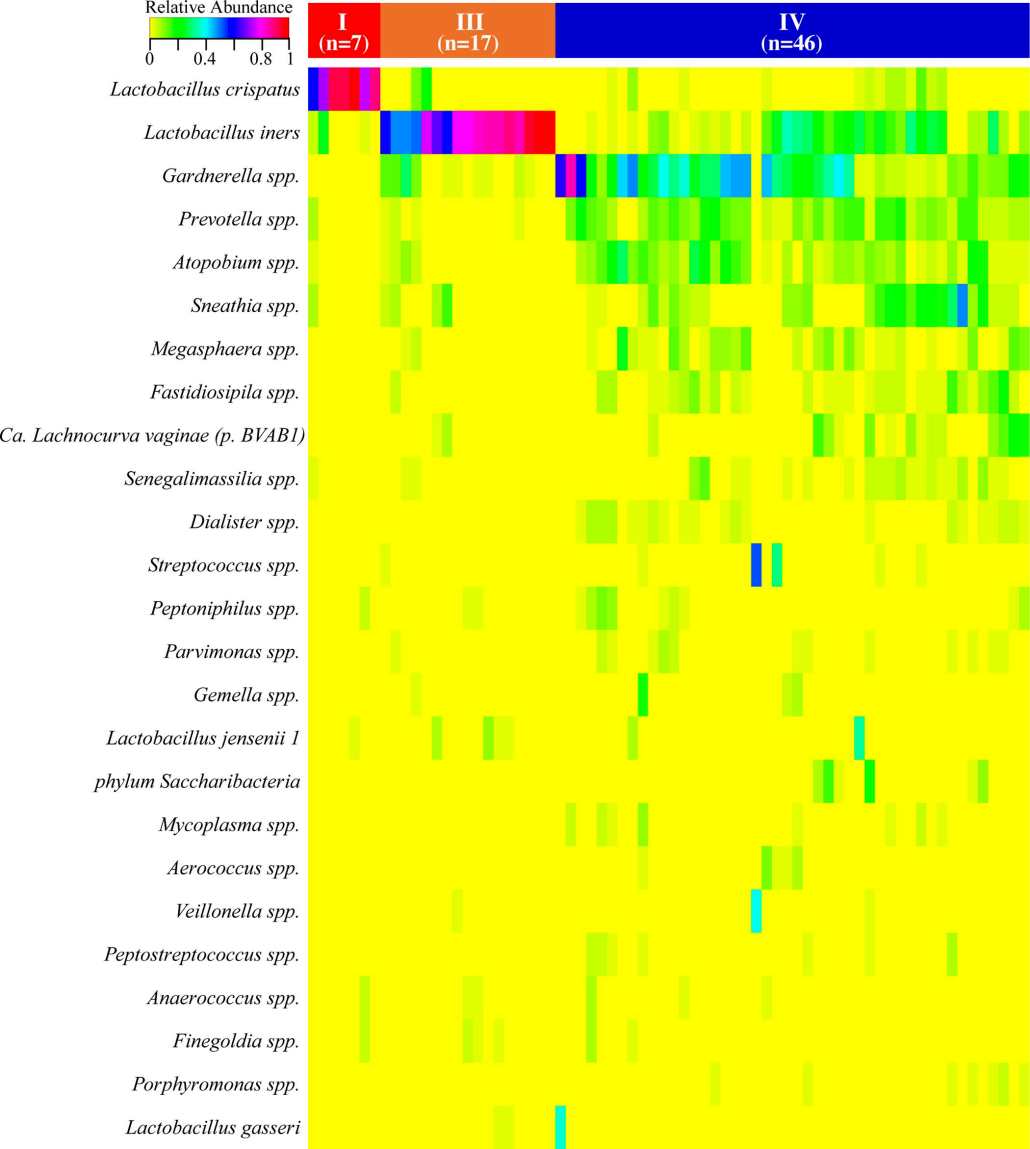
Heatmap displaying the relative abundance of the 25 most abundant bacterial taxa observed in the vaginal tracts of 70 American Indian women.

The vaginal microbiota of the seventy participants was characterized into three major vaginal community state types (CSTs), two of which were dominated by either *Lactobacillus crispatus* (CST I) or *L*. *iners* (CST III), and one comprised an abundance of anaerobes, including *Gardnerella*, *Prevotella*, *Atopobium*, and *Sneathia* species consistent with molecular-BV (CST IV). The frequency of CST varied significantly among the participants (Fig 1). Molecular-BV was most common with 66% of participants having CST IV microbiota, 24% having CST III, and 10% having CST I (Poisson regression, p-values <0.0001). None of the demographic variables listed in Table 1 were significantly associated with CST.

### Participants with molecular-BV had higher concentrations of BAs

We then quantified the concentration of common vaginal biogenic amines and their amino acid precursors (Table 2). All amino acids measured (arginine, lysine, ornithine, and tryptophan) were significantly higher in participants with CST I microbiota when compared to participants with either CST III or IV microbiota (all q-values <0.05). Participants with molecular-BV had higher concentrations of cadaverine, putrescine, trimethylamine, and tyramine compared to those with either CST I or III microbiota (q-values <0.05). Spermine and spermidine were higher in participants with CST I compared to participants with CST IV microbiota (q-values <0.05), but there was no significant difference between the concentrations observed in participants with CST III microbiota compared to those with either CST I or IV (q-values <0.05).

**Table 2:** Physiological concentrations of biogenic amines and amino acid precursors.

	**No. (%) detected in samples**		
	**CST I (7)**	**CST III (17)**	**CST IV (46)**
**Arginine (AA)**	7(100%)	17 (100%)	46 (100%)
**Lysine (AA)**	7 (100%)	17 (100%)	46 (100%)
**Ornithine (AA)**	7 (100%)	17 (100%)	46 (100%)
**Tryptophan (AA)**	7 (100%)	17 (100%)	46 (100%)
**Agmatine (BA)**	2 (28.6%)	6 (35.3%)	18 (39.1%)
**Cadaverine (BA)**	7 (100%)	17 (100%)	46 (100%)
**Putrescine (BA)**	7 (100%)	17 (100%)	46 (100%)
**Spermidine (BA)**	7 (100%)	17 (100%)	46 (100%)
**Spermine (BA)**	6 (85.7%)	10 (58.8%)	16 (34.8%)
**Trimethylamine (BA)**	7 (100%)	15 (88.2%)	38 (82.6%)
**Trimethylamine oxide (BA)**	7 (100%)	17 (100%)	46 (100%)
**Tyramine (BA)**	7 (100%)	17 (100%)	45 (97.8%)
	**Mean concentration (μM) (range)**		
**Arginine (AA)**	301.5 (93.9–715.3)^b^^,^^c^	158.5 (4.3–490.7)^b^	37.8 (0.32–328.08)^a^
**Lysine (AA)**	1013.2 (219.03–1700.5)^b^^,^^c^	422.5 (50.6–3558.1)	245.5 (3.1–1783.7)^a^
**Ornithine (AA)**	1403.5 (48.8–2994.3)^b^^,^^c^	127.9 (16.9–661.7)	129.5 (1.7–1256.9)^a^
**Tryptophan (AA)**	142.8 (39–275.4)^b^^,^^c^	50.1 (8.3–102.8)^b^	37.8 (0.3–212.6)^a^
**Agmatine (BA)**	0.36 (0.29–0.4)	0.4 (0.17–0.7)	1.3 (0.2–13.5)
**Cadaverine (BA)**	128.8 (12.7–425.1)^b^	163.8 (7.5–688.5)^b^	6170.4 (5.7–129384.5)^a^
**Putrescine (BA)**	392.9 (31.1–2033.2)	345.9 (15.3–1210.7)^b^	1167.6 (15.8–7079.2)^a^
**Spermidine (BA)**	81.6 (40.7–151.9)^b^^,^^c^	36.5 (1.2–101.3)	32.3 (0.7–162.1)
**Spermine (BA)**	629.34 (76.3–1866.8)^b^	138.3 (0.9–425.1)	216.2 (0.5–1977.7)^a^
**Trimethylamine (BA)**	18.4 (1.8–71.2)^b^	68.3 (2.2–427.9)^b^	375.8 (2.4–1959.3)^a^
**Trimethylamine oxide (BA)**	17.6 (9.3–28.3)^b^	38.3 (0.3–194.1)^b^	11.7 (0.1–102.4)^a^
**Tyramine (BA)**	6.2 (0.4–20.3)^b^	79.1 (0.5–416.8)	140.4 (0.3–863.2)^a^

These values reflect non-imputed data; p-values estimated using pairwise t-tests, corrected using FDR.

^a^ indicates q <0.05 versus concentration in all other combined groups.

^b^ indicates q <0.05 compared to CST IV.

^c^ indicates q <0.05 compared to CST III.

### Association between CST and participant behavioral and sexual practices

We next assessed the association between participant behavioral and sexual health practices with CST (Table 3). Of the 54 (79%) participants that identified as smokers, most (66%) had molecular-BV compared to 25% with CST III microbiota. Of the 18 (26%) participants that reported a history of douching, 78% had molecular-BV compared to those with CST III (17%) or CST I microbiota (5%). However, these proportions were not significantly different.

**Table 3:** Participant lifestyle and sexual practices.

Participant Details N (%)	CST I 7 (10%)	CST III 17 (24%)	CST IV 46 (66%)	Total Participants N = 70
**Smoking Status**				
Smoker, 1–5 cigarettes / day	2 (28.6)	7 (41.2)	19 (41.3)	28 (40)
Smoker, 6–10 cigarettes/ day	1 (14.3)	3 (17.6)	8 (17.4)	12 (17.1)
Smoker, 11–20 cigarettes / day	2 (28.6)	3 (17.6)	9 (19.6)	14 (20)
Non-Smoker	2 (28.6)	4 (23.5)	10 (21.7)	16 (22.9)
**Do you douche**				
Yes	1 (14.3)^b^	3 (17.7)^b^	14 (30.4)^a^	18 (25.7)
**Currently Sexually Active with a partner**				
Yes, partner (boyfriend/girlfriend)	5 (71.4)^b^	9 (52.9)^b^	32 (69.6)^a^	46 (65.7)
Yes, friend/acquaintance/ hook-up	2 (28.6)	2 (11.8)	3 (6.5)	7 (10)
Yes, spouse	0 (0)	6 (35.3)	5 (10.9)	11 (15.7)
No	0 (0)	0 (0)	6 (13)	6 (8.6)
**What gender do you usually have intercourse with?**				
Men only or primarily	7 (100)	16 (94.1)	45 (97.8)	68 (97.1)
Women only or primarily	0 (0)	1 (5.9)	1 (2.2)	2 (2.9)
**Partners in the last month**				
0	2 (28.6)	0 (0)	6 (13)	8 (11.4)
1	3 (42.9)^b^^,^^c^	15 (88.2)^b^	35 (76.1)^a^	53 (75.7)
2+	2 (28.6)	2 (11.8)	5 (10.9)	9 (12.9)
**Alcohol and/or drugs involved in last sexual encounter**				
Yes, Partner and Self	3 (42.9)^b^	2 (11.8)^b^	12 (26.1)	17 (24.3)
Yes, Partner	0 (0)	2 (11.8)	3 (6.5)	5 (7.1)
Yes, prefer not to say	1 (14.3)	0 (0)	3 (6.5)	4 (5.7)
No	3 (42.9)^b^^,^^c^	13 (76.5)^b^	28 (60.9)^a^	44 (62.9)
**Partner suspected/known to use needles**				
Yes	3 (42.9)^b^	3 (17.6)^b^	12 (26.1)	18 (25.7)
**Partner suspected/known to have Hepatitis C**				
Yes	4 (57.1)	0 (0)	10 (21.7)	14 (20)
**Partner suspected/known to have STI**				
Yes	2 (28.6)	1 (5.9)	10 (21.7)	13 (18.6)
**Partner suspected/known to be non-monogamous**				
Yes	3 (42.9)^b^	6 (35.3)^b^	20 (43.5)^a^	29 (41.4)

P-values estimated using pairwise two-sample tests for proportions with continuity correction; corrected for multiple comparisons using FDR.

^a^ indicates q <0.03 compared to combined group of CST I and III.

^b^ indicates q<0.03 compared to CST IV.

^c^ indicates q<0.03 compared to CST III.

Most participants (97%) reported that they only, or primarily, engaged in intercourse with men, with zero participants reporting that they engaged in sex with both men and women. All but six participants reported that that they were currently sexually active (defined as engaging in vaginal, oral, or anal intercourse). Most participants (66%) reported engaging in intercourse with a partner (not a spouse), and having had one sexual partner in the last month (76%). On average, participants reported having had intercourse 9 times (range 0–60 times) within the last month. Twenty-six participants (37%) reported the use of either alcohol or drugs (or a combination) either by (7%) or with (65%) their partner during their last sexual encounter. Eighteen participants (26%) reported that their partner was suspected or known to use needles and 29 participants (41%) reported that their partner was suspected or known to be non-monogamous. The frequency of CST varied among those that reported alcohol and/or drugs being involved in the last sexual encounter, and among those that reported their partner was suspected or known to either use needles or be non-monogamous, with molecular-BV being most frequent compared to either CST I or CST III (q-values <0.03). In models unadjusted and adjusted for CST, none of the variables listed in Table 3 were significantly associated with the measured vaginal metabolites.

### Association between psychosocial stress, vaginal microbiota, and vaginal metabolites

**Fig 2:**
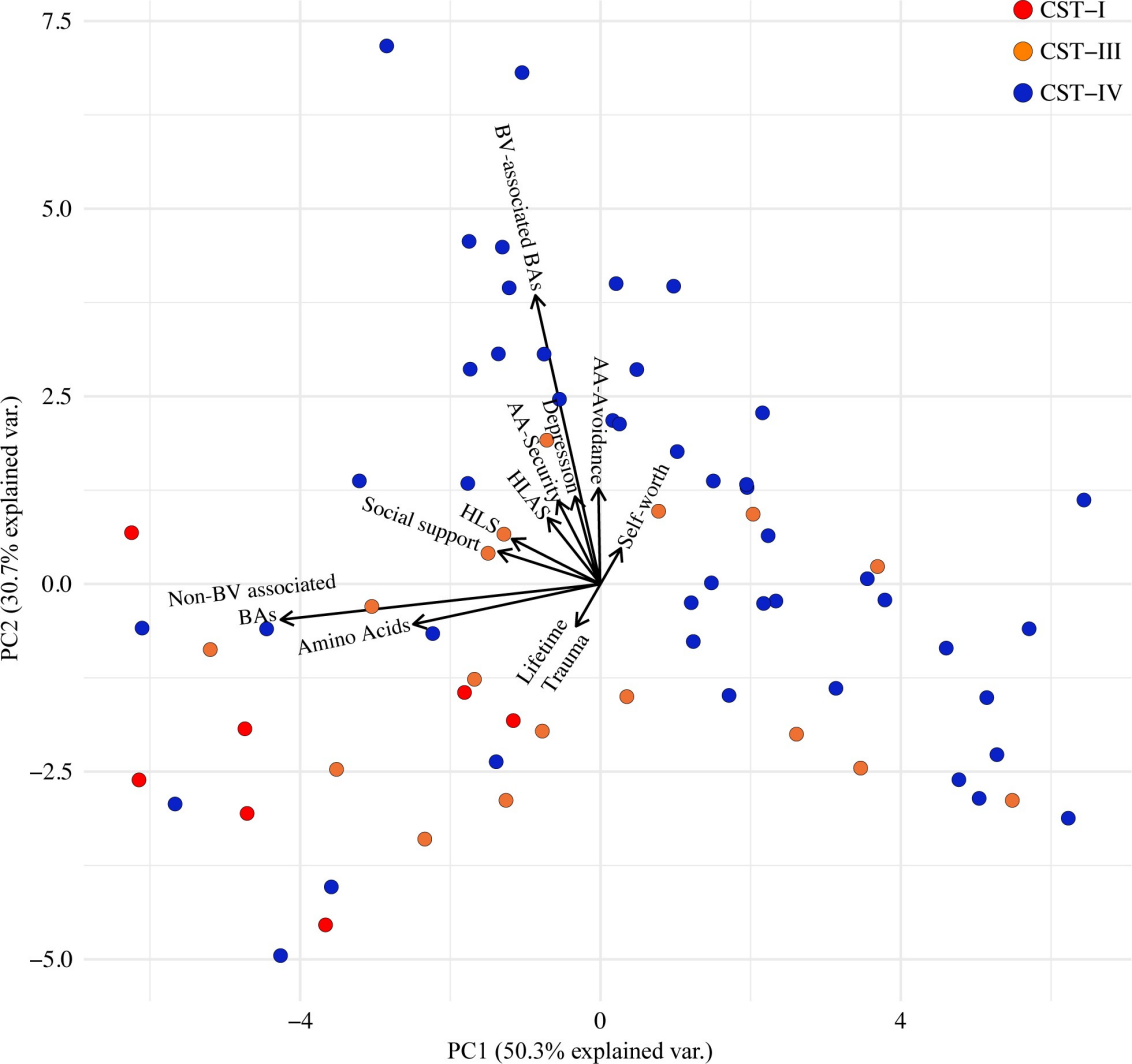
Associations of measures of psychosocial stress, the vaginal microbiota, and vaginal metabolites PCA biplot. Circles represent individuals color coded by CST and black arrows indicate the two principal component loading vectors.

We next interrogated the relationship between measures of psychosocial stress (adult attachment, social support, self-worth, depression, lifetime trauma, historical loss, and historical loss associated symptoms) with the vaginal metabolites and vaginal microbiota. An exploratory PCA of vaginal metabolites and measures of psychosocial stress indicated that participants with molecular-BV primarily clustered together (Fig 2). The first component largely corresponded to the collective measures of all amino acids and the non-BV associated BAs (spermidine and spermine), which were corelated with each other. The second component largely consisted of the collected measure of the BV-associated BAs (agmatine, cadaverine, putrescine, tyramine, trimethylamine, and trimethylamine oxide), as well as social support, historic loss, historic loss associated anxiety, the adult attachment measures of security and avoidance, and depression.

**Fig 3:**
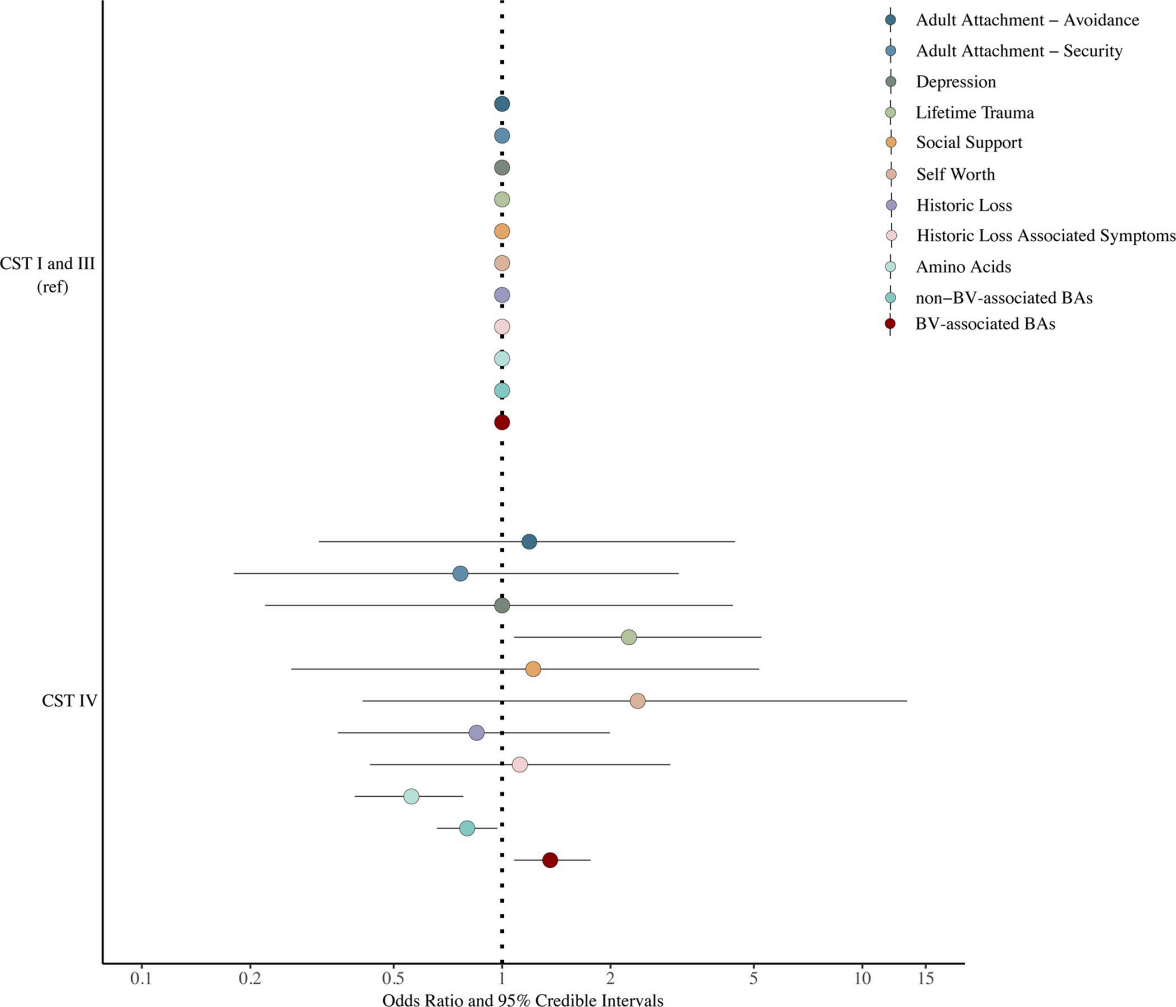
Adjusted odds ratio for CST by measures of psychosocial stress. Circles represent point estimates while bars indicate 95% credible intervals. Odds ratios were adjusted for smoking status.

Bayesian multinomial logistic regression modeling adjusted and unadjusted for smoking status indicated that higher levels in BV-associated BAs and lifetime trauma were associated with increased odds of having molecular-BV compared to a grouped category of participants with either CST I or III microbiota (Fig 3) (BV-associated BAs adjusted odds ratio (aOR): 1.36, 95% credible interval (CrI): 1.1–1.8; lifetime trauma aOR: 2.3, 95% CrI: 1.1–5.25). Higher levels of amino acids and non-BV-associated BAs (spermine and spermidine) were associated with decreased odds of having molecular-BV compared to participants with either CST I or III microbiota (non-BV associated BAs aOR: 0.8, 95% CrI: 0.6–0.97; Amino Acid aOR: 0.56, 95% CrI: 0.39–0.78).

### Measures of psychosocial stress are associated with lifestyle and sexual practices

**Fig 4:**
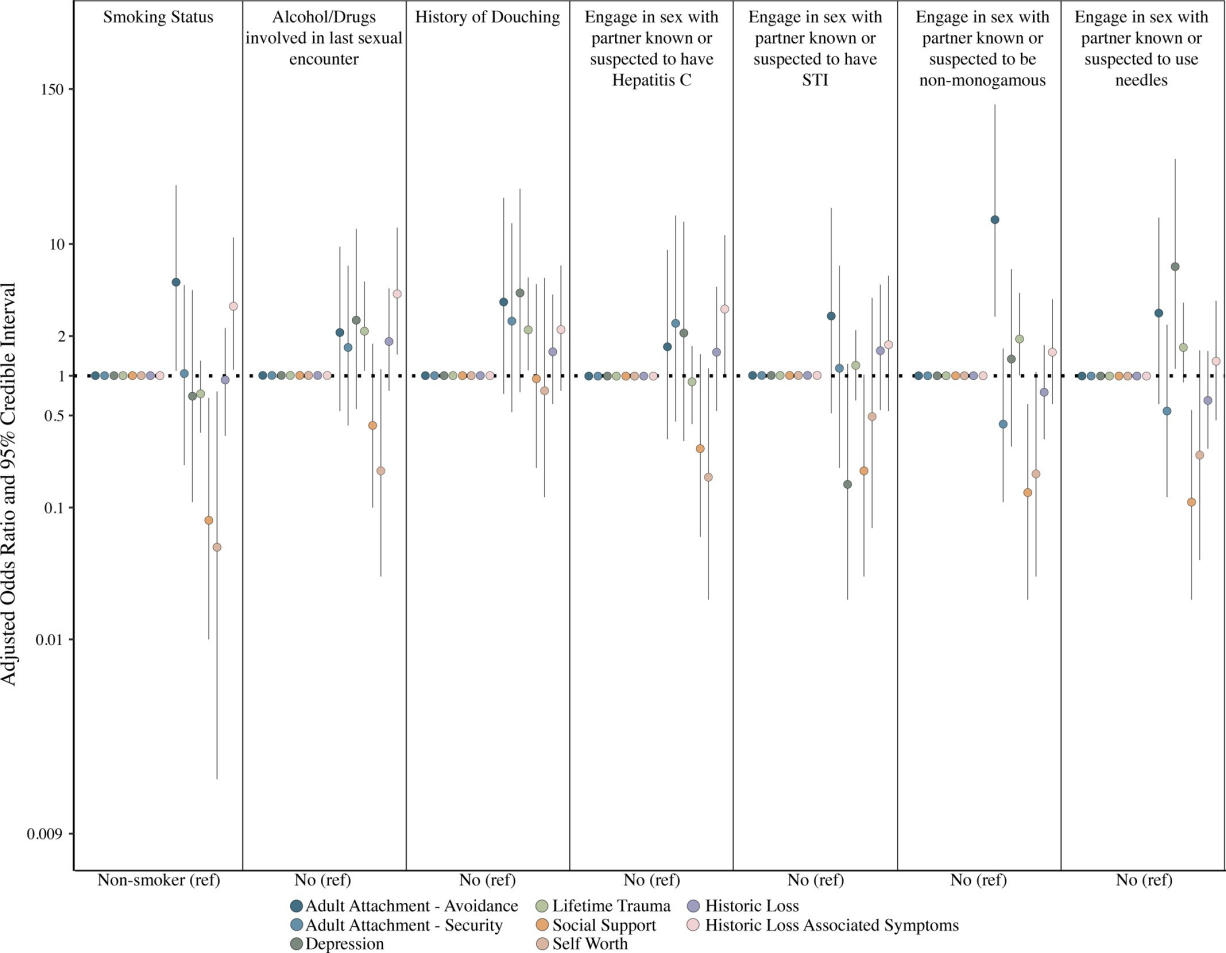
Adjusted odds ratio for sexual and lifestyle practices by measures of psychosocial stress. Circles represent point estimates while bars indicate 95% credible intervals.

We next evaluated the associations between measures of psychosocial stress with lifestyle and sexual practices (Fig 4). Unadjusted Bayesian regression analysis indicated that participants with greater social support scores were less likely to report having been with a partner known or suspected to use needles (aOR: 0.11, 95% CrI: 0.02–0.55) or known or suspected to be non-monogamous (aOR: 0.13, 95% CrI: 0.02–0.61). Both greater social support and self-worth scores were significantly associated with decreased odds of reporting as a smoker. Participants with high historic loss associated symptoms (HLAS) and adult attachment-avoidance scores were significantly associated with greater odds of being a smoker (HLAS aOR: 3.37, 95% CrI: 1.1–11.2; adult attachment-avoidance aOR: 5.1, 95% CrI:1.1–27.9). High scores of adult attachment-avoidance were also significantly associated with reporting having had intercourse with a non-monogamous partner (aOR: 15.3, 95% CrI: 2.8–114.3) within the past year, while participants with higher scores of lifetime trauma and HLAS were more likely to report drugs and/or alcohol being used in last sexual encounter (Lifetime trauma aOR: 2.2, 95% CrI: 1.1–5.1; HLAS aOR: 4.2, 95% CrI: 1.4–13.3). Notably, high scores of lifetime trauma were associated with douching (aOR: 2.23, 95% cRI: 1.1–5.57).

### Association between measures of psychosocial stress and vaginal metabolites

The association between measures of psychosocial stress and select vaginal metabolites were evaluated using Bayesian regression and adjusting for CST and smoking status given their independent associations with these metabolites (S1 Table). For every one-unit increase in social support scores, participants had higher concentrations of lysine (adjusted fold change (aFC): 4.5, 95% CrI: 1.3–15.9). Similarly, every one-unit increase in adult attachment-security scores was associated with higher concentrations of tryptophan (aFC: 4.5, 95% CrI: 1.5–14.1). Finally, every one-unit increase in self-worth score was associated with lower concentrations of trimethylamine oxide (aFC: 0.1, 95% CrI: 0.02–0.69). High scores of historic loss were also significantly associated with cadaverine (aFC: 3.1, 95% CrI: 0.97–9.7). Notably, higher scores of lifetime trauma were associated with higher concentrations of spermine (aFC: 3.3, 95% CrI: 1.2–9.2).

### Measurement model

Finally, we evaluated the relationship between the three identified latent variables, historic loss (HLS) associated anxiety/depression, interpersonal reliance, and vaginal BAs with the dependent variable (molecular-BV vs. a grouped category of participants with either CST I or III microbiota). The measurement model (S1 Fig) had an adequate fit to the data, with a Tucker-Lewis Index of 0.967, a comparative fit index of 0.977, a root-mean-square error of approximation of 0.057, and an insignificant chi-squared test of model fit (χ^2^ = 39.3, df = 32, p = 0.174). Standardized factor loadings for all three latent variables were statistically significant and averaged >0.7 for each construct. The largest correlation was between interpersonal dependency and the historical loss associated anxiety/depression construct (-0.299, p = 0.017). The correlations between the vaginal BA construct, the historical loss associated anxiety/depression construct, and the interpersonal reliance construct, were positive but relatively smaller (<0.2).

### Path diagram and direct effect estimates of the final SEM

**Fig 5:**
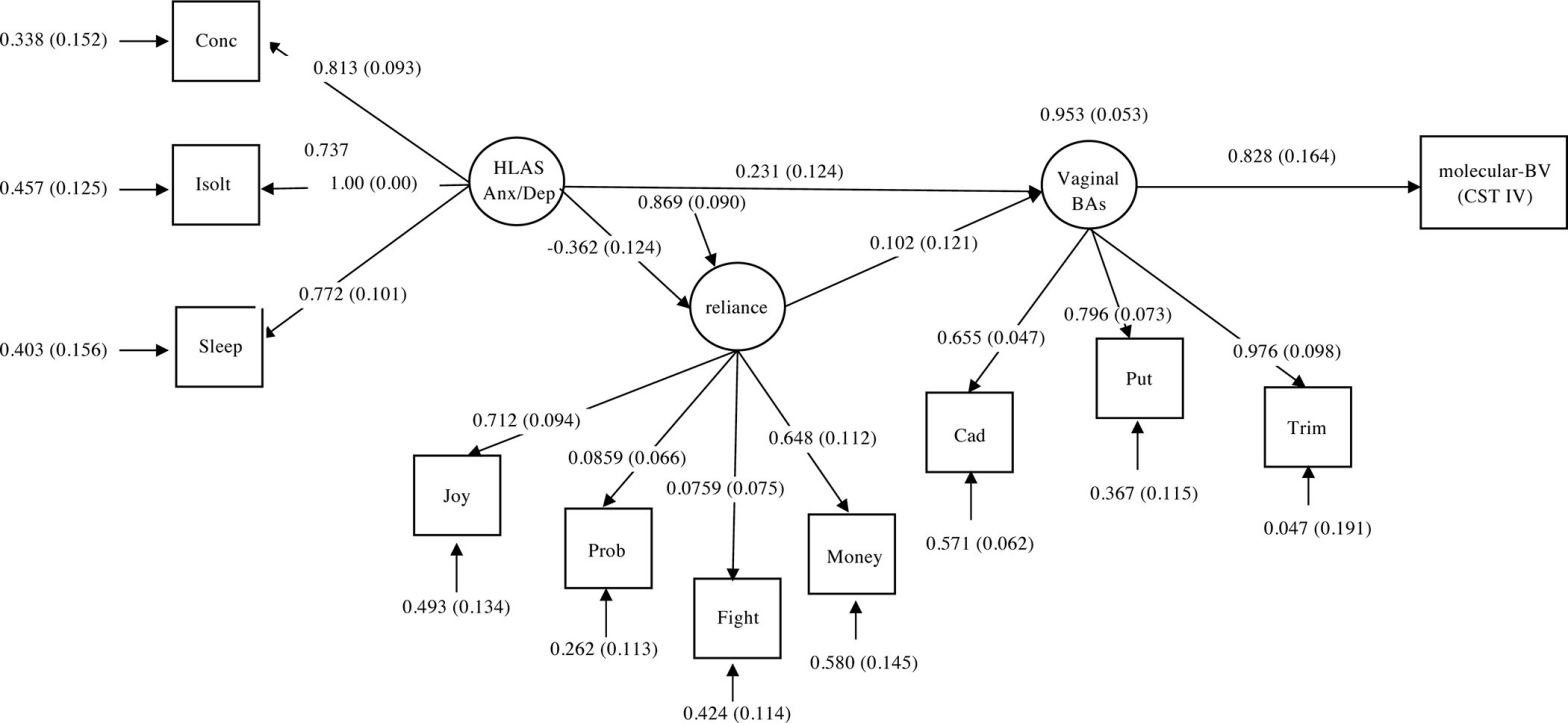
Structural equation model (SEM) with standardized results. Pathway to molecular-BV (CST IV) through interpersonal reliance and vaginal biogenic amines.

The pathways from the HLS associated anxiety/depression construct, the interpersonal reliance construct, the vaginal biogenic amines construct, and CST-IV status are shown in Fig 5. The overall fit of the model was χ^2^ = 32.97, df = 41, p = 0.81 with a comparative fit index of 1.0, a Tucker-Lewis Index of 1.2, and a root-mean-square error of approximation of 0.00. The extreme fit values reflect the small sample size (n = 70). An increase in the vaginal biogenic amine construct score was appreciably related to an increase in the probability of having molecular-BV (effect (E) = 0.83, standard error (SE) = 0.16). The interpersonal dependency construct did not appear to have any notable effect on the vaginal biogenic amines construct but the HLS associated anxiety/depression construct showed an effect on both the interpersonal dependency construct (E = -0.36, SE = 0.12) and the vaginal biogenic amines construct (E = 0.23, SE = 0.12). The total indirect effect of HLS associated anxiety on CST-IV status was 0.16 (SE = 0.095).

## Discussion

It is well-established that the vaginal microbiota play an important role in mediating female reproductive and sexual health [89–91]. In 2011, Ravel et al. observed that the vaginal microbiota of U.S. reproductive-aged women clustered broadly into five community state types (CSTs) and demonstrated that the prevalence of each CST varied across race and ethnicities [91]. In a study of nearly 400 U.S women, CST IV/molecular-BV microbiota was most prevalent within Hispanic (38%) and non-Hispanic Black (40%) women compared to non-Hispanic white (10%) and Asian (20%) women [91]. Consistently, this study reveals that the majority of AI participants were also dominated by CST IV/molecular-BV (66%)—the highest reported prevalence within a given population, to date. This high rate of molecular-BV corresponds to the higher observed rates of STIs among AI populations [1], and is consistent with findings that BV is associated with increased risk for STIs [68, 92–94].

The reasons for the racial disparities in molecular-BV prevalence are not clear, with differences in sexual and hygienic behaviors having previously been proposed [95–98]. Hygienic practices such as douching and individual behaviors such as the involvement of drugs and alcohol during the last sexual encounter, and condom use have been shown to vary by race and socioeconomic status and to increase odds of BV [99–101]. Consistently, we observed a history of douching, the involvement of drugs and/or alcohol during the last sexual encounter, partner suspected or known to use needles, and partner concurrency to be significantly associated with molecular-BV (CST-IV microbiota). These data support emerging evidence that points towards a need to investigate the association between sexual networks, of which partner concurrency is a marker, and STI and BV prevalence in AI populations [13, 102]. It is critical to acknowledge that previous studies have found that both individual-level sexual practices and sociodemographic factors do not fully account for observed disparities in STIs among other minority populations [1, 11–13, 103–105] and thus may not fully account for the discrepancies in molecular-BV observed herein.

Our data and others suggest that differences in perceived stress may play a mediating role in development of BV [22, 46, 106, 107]. The effect of stress may enrich for this non-optimal CST by impairing the host immune response [106, 107], inhibiting the deposition of glycogen [106], or by increasing sexual practices [107], such as having sex while influenced by alcohol or drugs [108]. Consistently, we observed that measures of psychosocial stress were associated with behavioral practices such as smoking, douching, and having sex with partners suspected or known to use needles. A large percentage of our participants reported the involvement of drugs or alcohol during their last sexual encounter (37.1%), of which 70% had CST IV microbiota. Further, most of these participants also had high scores of historic loss associated symptoms and a history of smoking. Smoking itself has been shown to have a dose-dependent association with both CST-IV and BV [109] and among adolescent and reproductive-age women, ‘coping with stress’ and ‘stress relief’ are the most commonly cited motives for smoking [110–113]. Among African American women, smoking has been associated with the frequency and perceptions of overall race-related, individual, and cultural race-related stress [23].

One measure of psychosocial stress, lifetime trauma was associated with molecular-BV. Further, several measures of psychosocial stress, including HLAS and lifetime trauma were associated with sexual and lifestyle behaviors, including smoking and partner concurrency. Further, the HLS anxiety construct had a positive effect on the vaginal biogenic amine construct comprised of putrescine, cadaverine and trimethylamine [55, 61, 114, 115]; and importantly, we observed associations between psychosocial stress and BV-associated BAs or their precursors. This is important as it identifies specific risk factors directly related to BAs, and indirectly to molecular-BV, and along with the observed high concentrations of these metabolites in women with molecular-BV compared to CST I or CST III women, is consistent with previously reported literature [62–64, 78, 80, 116].

Conventionally thought of as biomarkers of BV [63, 117], these biogenic amines are generally produced from bacterial amino acid decarboxylation reactions in the presence of acid stress [62, 63, 76, 118]. These reactions are known to decrease environmental acidity, and are hypothesized to promote the colonization and outgrowth of BV-associated bacteria [63, 76]. Further, biogenic amines have been shown to increase pathogen virulence [76, 118], protect pathogens, including *Neisseria gonorrhoeae*, against innate immune defenses [84, 119], and have been associated with infections of the most prevalent bacterial and viral STIs [79, 80]. Several studies have demonstrated increases in levels of BAs in response to stressful stimuli [85, 87, 88, 120]. Conversely, spermidine and spermine tend to be associated with ameliorative effects against stressful stimuli [88], and some research indicates that diets rich in spermidine and spermine may enhance mitochondrial function and autophagy within the brain thereby stave-off age-related cognitive decline and may calm neuroinflammatory responses [121–123]. This may explain the positive association we observed between lifetime trauma and spermine. While we hypothesize psychosocial stress impacts vaginal metabolites, it is likely multi-faceted as behavioral practices such as smoking have been previously associated with BAs [78]. It is possible that, in addition to measures of psychosocial stress, other behavioral and sexual practices affect the BAs leading to a vaginal environment less optimal for vaginal lactobacilli and at increased susceptibility to STIs. However, the hypothesized biological mechanism is currently unknown, and mechanistic studies designed to establish the relationship between psychosocial stress, vaginal metabolites, and vaginal bacteria are merited.

There are several limitations to our study. First, while this is the first study characterizing the vaginal microbiota of AI women, our sample size is relatively small, limiting our power to detect differences among some variables. This is particularly evident in our unadjusted analysis of participant measures, as we visually observe differences in distributions across variables, but while approaching statistical significance, they did not meet the criteria of *α* = 0.05. This also hindered our ability to have adequate representation across factors (such as CSTs I and III). Secondly, we are limited by the cross-sectional and self-reported nature of our data; therefore, we cannot make causal inferences regarding the impact on stress upon the vaginal microbiota, nor can we capture short term fluctuations of the metabolites or microbiota. Further, the data described herein may not be representative of other tribal communities or sovereign nations and must be interpreted in context of Northwestern Plains American Indian Populations. It is critical to note that although we identified important variables within our model, this does not preclude the value of other variables. Finally, we did not measure biological stress variables, such as cortisol, and it is possible that individuals may have under-reported or underestimated their stress.

In summary, we were able to describe the frequency of molecular-BV among reproductive-aged AI women, identify the association between measures of psychosocial stress and lifestyle and sexual practices upon the vaginal microbiota and select vaginal metabolites.

## Methods

### Positionality statement

This research was conducted by Indigenous and non-Indigenous scholars from universities throughout the United States and the tribal college on the reservation where the study took place.

### Study setting

This study took place on a reservation in a western state in the Northern Plains region of the United States. There are nearly as estimated 6,000 enrolled tribal members living on the reservation who are descendants of the Nakoda, Nakota, Nakona, Lakota, and Dakota Nations.

### Ethics statement

This study, including the recruitment of participants and research presented herein, builds upon a 14-year partnership between tribal members and researchers affiliated with Montana State University (MSU), as previously described [124–132]. Briefly, this collaborative partnership utilizes a community based participatory research framework to combine Indigenous expertise in traditional knowledge, contemporary reservation culture, and local tribal resources with Westernized research skills in sexual and reproductive health research among tribal members. In the United States, American Indian tribes have the legal right to regulate all affairs in their tribal community, including research and the legal right of sovereignty and governance over data conducted from community members. In the sovereign nation described herein, the tribal Institutional Review Board (IRB) oversees and makes decisions about research and data as directed by the Tribal Executive Board. The Tribal IRB approved the undertaking of this research and its publication, as indicated, with the requirement that neither the reservation nor tribes be explicitly named. A community advisory board (CAB) made up of five tribal members provided oversight and guidance on the study, including development of the survey and data collection instruments, study design, and interpretation of the qualitative and quantitative data. The CAB and the IRB in the tribal community where the study took place provided ethical approval for the study, reviewed, and approved the language within the manuscript for publication prior to submitting (FWA00019355, approved 08/11/16). The MSU IRB also provided ethical oversight, guidance, and approval for this study (FWA00000165, approved 09/08/16). All participants provided written informed consent. All research was conducted in compliance with relevant guidelines, and regulations.

### Population and sampling information

This is a cross-sectional study that primarily used word-of-mouth to recruit Northwestern Plains American Indian women in 2016. Networking through agencies on the reservation was also used. For confidentiality purposes, we use the general descriptors of Northern Plains rather than specific tribal names. Eligibility for participation was restricted to include enrolled tribal members who were 18–45 years of age and having a menstrual cycle. Participants were ineligible be involved if they were pregnant or reported use of antibiotics in the prior three months, a total of 70 participants were recruited. This study did not assess gender or sexual identity. Participants were asked to provide detailed information regarding sexual preferences, habits, and practices, including the gender that they usually engage in sex with, and to disclose patterns and concerns experienced with their partner (e.g., whether a partner was jealous a lot of the time); however, the gender of the partner was left ambiguous. Following recruitment, participants were surveyed in a one-on-one interview with a trained social scientist who was assigned female at birth (survey described below). Prior to administration of the survey, participants were informed that this could elicit physiological stress and were advised that they do not have to participate and could rescind consent at any time. At the completion of the interview, participants were provided instructions and a private stall to self-collect mid-vaginal swabs that were immediately frozen at -20°C before being moved to -80°C storage within 6 hours. Samples remained stored at -80°C until use. All participants received $100 VISA gift card on completion of the study.

### Survey measures

The interview followed a modified version of the American Indian Service Utilization, Psychiatric Epidemiology, Risk and Protective Factors Project (AI-SUPERPFP) survey [133] (S1 File), an in-depth quantitative and qualitative survey covering demographics, perceived psychosocial stress and stressors, gynecological health, sexual behavior practices, and known STI and BV risk factors. Measures obtained from the survey and used in this analysis are described below:

#### Self-worth

Self-worth was measured using a series of six questions adapted from the Rosenberg Self-Esteem Scale [134] to assess global self-esteem. Responses were provided on a 5-point Likert Scale ranging from (1) *disagree* to (5) *agree*. An overall *Self-worth* score was calculated for each participant by reverse-coding negative Likert questions and summing responses across all questions, where higher scores indicate high self-worth.

#### Adult attachment

Adult attachment was measured using the Measure of Attachment Qualities (MAQ) Instrument Scale [135] which consisted of 14 questions and included subscales to evaluate 1) secure attachment and 2) avoidance. Responses were provided on a 4-point scale ranging from (1) *disagree* to (4) *agree*. Scores for each subscale were calculated by reverse-coding negative responses and summing the responses per participant, where higher scores indicate secure attachment tendencies or relationship avoidance tendencies.

#### Social support

Social support was measuring using the Multidimensional Scale of Perceived Social Support (MSPSS) [136]. The MSPSS is a validated scale that asks participants 12 questions about their perceived social support (i.e., emotional support, comfort, and assistance in making decisions) from family, friends, or significant others. To evaluate whether there was an association between social support upon the vaginal microbiota and vaginal BAs, we reverse-coded negative Likert questions, and calculated a total *Social Support Score* per participant, where higher scores indicate more social support. For the CFA and SEM, a latent factor, *interpersonal reliance*, was identified during preliminary psychometric tests, and was parceled to include four items: 1) *I have friends with whom I can share my joys and sorrows; 2) I can talk about my problems with my friends; 3) if someone wants to fight me*, *my family will stand by* me; and 4) *there is someone I can borrow money from in an emergency*. This latent factor was included in the CFA with the assumption that interpersonal reliance could potentially mitigate effects of anxiety and depression.

#### Depression

Depression was assessed by an adapted Center for Epidemiological Studies Depression Scale [137, 138]. This scale assessed nine symptoms with responses ranging from 1) *never or rarely* to 4) *most of the time*, *or all of the time*. A total depression score was calculated by reverse-coding negative responses and summing the responses per participant; higher scores indicate higher scores of depression.

#### Lifetime trauma

Adverse life events were measured using a 28-question construct which assesses various types of exposure to stressors including verbal and emotional abuse, physical injuries, and suicide. Each response was scored dichotomously (0 = unexposed, 1 = exposed). An overall summary score, representing the total of number of adverse life events, was calculated where higher scores indicating greater exposure to lifetime trauma.

#### Historical loss and historical loss associated symptoms

In addition to the measures described above, the frequency with which participants think about historical loss (loss of culture, land, and people as a result of colonization) was measured using the Historical Loss Scale (HLS) [139], which consists of 12 questions with responses provided on a 6-point frequency-based Likert scale, ranging from (1) *never* to (6) *several times a day*. The frequency by which participants experience particular symptoms associated with historical loss was measured using the Historical Loss Associated Symptoms (HLAS) scale [139]. The HLAS scale comprises 12 questions with responses provided on a 5-point scale ranging from (1) *never* to (5) *always*. Higher scores of both scales indicate a participant self-reported thinking about HLS or being affected by HLAS often.

### Taxonomic assignment and community state type (CST) profiling

DNA extraction, PCR amplification and sequencing of the 16S rRNA gene V3-V4 and bioinformatics processing using the *dada2* pipeline [140] followed the methods described by Holm et al. 2018 [141]. Taxonomic assignments were performed using the RDP Classifier trained with SILVA (version 128) and speciation of specific vaginal bacteria with SpeciateIT (ravel-lab.org/speciateit). For each sample, CST classifications were assigned as previously described, using Jensen-Shannon divergence and Ward linkage hierarchical clustering [90, 91].

### Sample preparation for metabolomics

Samples were eluted from Copan Nylon Flocked dry swabs using 70% methanol and analyzed with the 5500 QTRAP LC/MS/MS system (Sciex, Framingham, MA) in Metabolomics Lab of Roy J. Carver Biotechnology Center, University of Illinois at Urbana-Champaign. Software Analyst 1.6.2 was used for data acquisition and analysis. The 1200 series HPLC system (Agilent Technologies, Santa Clara, CA) includes a degasser, an autosampler, and a binary pump. The LC separation was performed on an Agilent Eclipse XDB-C18 (4.6 x 150mm, 5μm) with mobile phase A (0.1% formic acid in water) and mobile phase B (0.1% formic acid in acetonitrile). The flow rate was 0.4 mL/min. The linear gradient was as follows: 0-3min, 95%A; 9-13min, 5%A; 13.5-18min, 95%A. The autosampler was set at 10°C. The injection volume was 5 μL. Mass spectra were acquired under positive electrospray ionization (ESI) with the ion spray voltage of +5000 V. The source temperature was 500°C. The curtain gas, ion source gas 1, and ion source gas 2 were 33, 65, and 55 psi, respectively. Multiple reaction monitoring (MRM) was used for quantitation: cadaverine (m/z 103.0 → m/z 69.0); putrescine (m/z 189.0 → m/z 30.0); spermine (m/z 199.9 → m/z 91.1); spermidine (m/z 146.0 → m/z 30.0); and tyramine (m/z 138.1 → m/z 77.1). The levels of detection for each metabolite were as follows: cadaverine (100nM), putrescine (100nM), spermidine (20nM), spermine (20nM) and tyramine (5nM). For the purpose of plotting, “missing values” were imputed with one-half the minimum value obtained for a given metabolite [142–145].

### Statistical analysis

We sought to characterize the vaginal bacterial community composition of an American Indian population and to evaluate associations between behavioral, demographic, and measures of psychosocial stress with the vaginal microbiota and vaginal biogenic amines. Unadjusted associations were assessed using a two-sample test for equality of proportions with continuity correction in R Statistical Software (Tables 1 and 3) or a two-sample t-test (Table 2). An exploratory biplot showing the first two components of a principal component analysis (PCA) was constructed on scaled variables to visualize samples and the influence of variables. The PCA was conducted using the *prcomp* function and visualized using the *ggbiplot* package in R Statistical Software.

Bayesian multinomial logistic regression was utilized for modeling the association between measures of stress, high-risk behavior and vaginal CSTs as the data were too sparse for large-sample frequentist inference [146, 147]. Due to the small number of participants with CST I microbiota (n = 7), we created a combined reference category using participants with both CST I and CST III microbiota. When modeling the associations between the vaginal CSTs (outcome) and measures of psychosocial stress and vaginal metabolites (Fig 3), we performed unadjusted and adjusted analysis with only smoking was included as a covariate, and this is because smoking is known to be associated with the vaginal microbiota and because a large number of participants self-reported as smokers [109]. When modeling the association between sexual practices (outcome) and measures of psychosocial stress (Fig 4), we did not include any covariates. We also used Bayesian regression to model the association between measures of stress and select vaginal metabolites (response) while adjusting for CST and smoking status, and these covariates were included given their established relationship [78, 109].

For all models, estimation was carried out using Hamiltonian Monte Carlo [148–150] and its extension, the No-U-Turn (NUTS) Sampler [151]. We used weakly informative priors for the fixed and random effects (i.e., we did not impose any prior information on the estimates). Bayesian multinomial logistic regression was performed in the *brms* package [152] in R which runs RStan (Stan Development Team, 2015) in the background. Statistical significance was defined as Bayesian credible intervals for odds ratios excluding 1.

### Structural equation modeling (SEM)

Three latent variables were identified: HLAS anxiety and depression, interpersonal reliance, and the standardized values of three biogenic amines, trimethylamine, cadaverine, and putrescine. HLAS anxiety and depression was developed from a three-item solution with symptoms including: loss of concentration, feeling isolated or distant from other people when thinking about losses, and a loss of sleep. Spearman correlation analyses were performed to examine the associations between independent variables in the CFA and SEM. In the measurement model (S1 Fig), latent constructs were permitted to intercorrelate. SEM was used to explore relationships between HLAS of Anxiety/Depression, interpersonal dependency, and vaginal biogenic amines with the dependent variable (molecular-BV status versus non-molecular-BV status). We estimated parameters using linear regression coefficients for indicators with Gaussian distributions and probit regression to model effects on CST-IV status. We examined model fit using χ^2^, the comparative fit index, the Tucker-Lewis Index, and the root-mean-square error of approximation. To accept the model, the χ^2^ should be non-significant, the comparative fit index and Tucker-Lewis Index>0.95, and the root-mean-square error of approximation <0.08 [153, 154]. Given that this was a preliminary analysis conducted in a small sample, we focused on relative effect sizes and standard errors of the effects. Spearman’s correlation analysis was conducted in R Statistical Software. CFA and SEM modeling were conducted in Mplus v. 8, STATA 14 for data management, cleaning, and recoding. Results from the pathway analysis are shown in Fig 5.

## Supporting information

S1 Fig Confirmatory factor analysis (CFA). (TIF)

S1 Table Associations between measures of psychosocial stress and vaginal metabolites. (XLSX)

S1 File Survey. (PDF)
