# Supplementary figures and images for: Vaginal microbiota of American Indian women and associations with measures of psychosocial stress
Source: PLoS One. 2021 Dec 10;16(12):e0260813. doi: 10.1371/journal.pone.0260813 (PMC8664215; doi:10.1371/journal.pone.0260813)

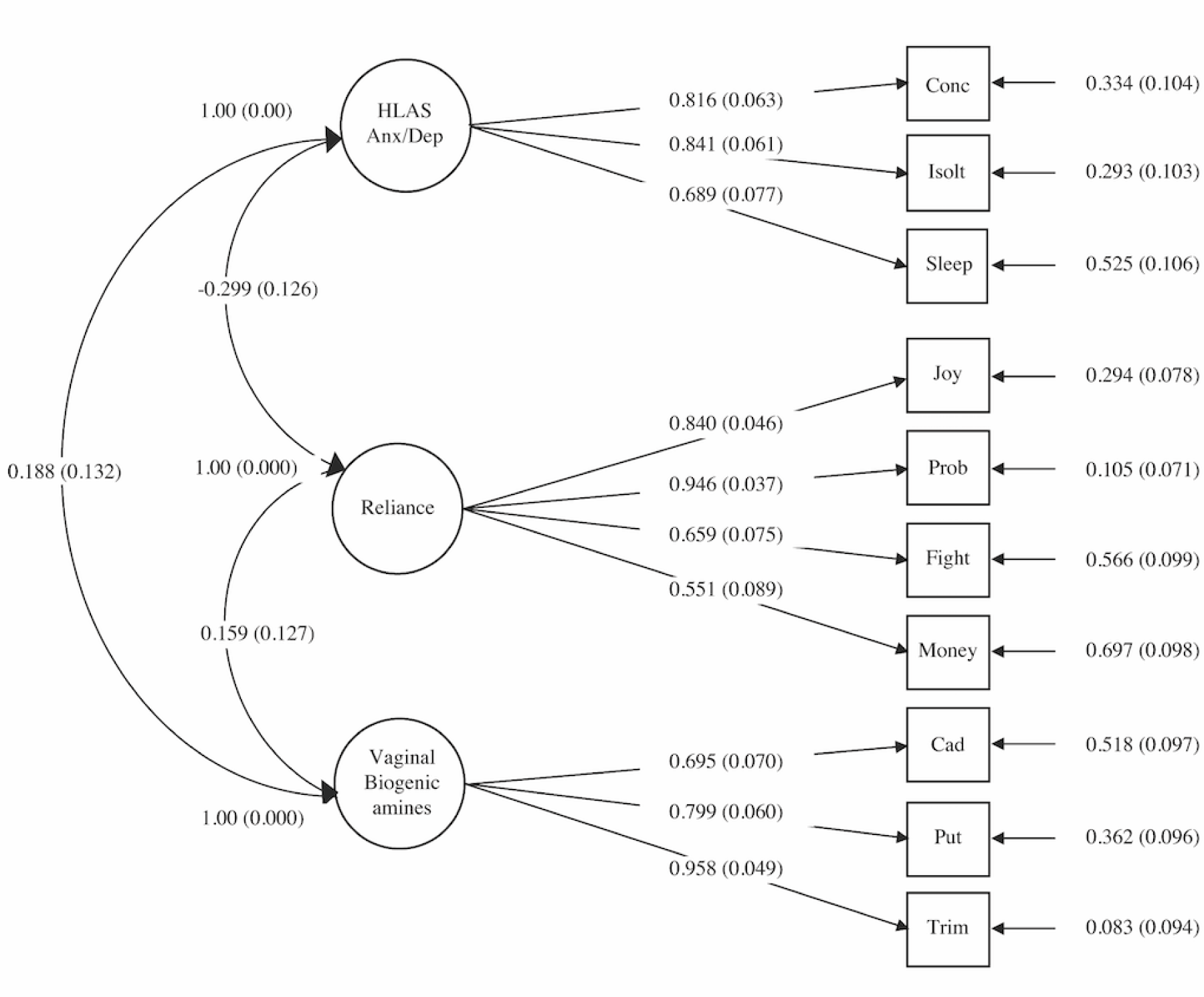

Supplement: S1 Fig — (TIF) [file pone.0260813.s001.tif]
