# Supplementary material for: Vaginal microbiota of American Indian women and associations with measures of psychosocial stress
Source: PLoS One. 2021 Dec 10;16(12):e0260813. doi: 10.1371/journal.pone.0260813 (PMC8664215; doi:10.1371/journal.pone.0260813)
Supplement: S1 File — (PDF) [file pone.0260813.s003.pdf]

INTERVIEW GUIDE

INTRODUCTION

Thank you for agreeing to talk with me today. We are interested in learning more about you. I will be asking you several questions about different things like your feelings, your intimate relationships and sex life as well as questions about some of your life experiences. I am asking you these questions because we are interested in how they may be influencing your microbiota (the bacteria that normally live inside your body) and your hormones (signals that your body uses to affect your bodily processes and mood). Because your hormones and microbiota affect your health, we want to learn what things we can do in day-to-day life to stay healthy. Some of the questions are personal and may make you feel uncomfortable. For example some people do not like talking about sex or alcohol and drug use. Please remember that you do not have to answer any questions that make you feel uncomfortable. Your participation in this project is very much appreciated and will be helpful for protecting your sexual and reproductive health, and the health of American Indians.

SECTION 1: DEMOGRAPHICS

The first several questions are general questions about you.

1.1 Where do you currently live? (Town) ____________________

1.2 Where did you grow up? ____

1.3 Are you enrolled in a tribe? YES NO

If yes which tribe are you an enrolled member of _______________

1.4 Are you an Associate member in a tribe? YES NO

If yes which tribe are you an Associate member in? ___________________

1.5 How old are you? __________________

1.6 What is the highest degree or level of school you have completed?

1 Grades 1-8 (Elementary)

2 Grades 9-12 (Some high school but no diploma)

3 Grade 12 or GED (High school graduate)

4 College 1 year to 4 years (Some college or technical school but no degree)

5 College 1 year to 4 years (Associate degree)

6 College 4 years (College degree: BA or BS)

7 Graduate degree (MA, MS, JD, MD, or PhD)

1.7 Which of these describes how you currently make a living? [check all that apply]

1. Student
2. Homemaker
3. Self-employed (including traditional crafts, Avon, etc.)
4. I have a job. (This includes the military.)
5. On public assistance -- AFDC, food stamps, SSI, GA, unemployment
6. I am not working, but I am looking for work.
7. I am not working, and I am not looking for work.
8. Other (Please write in.)____

SECTION 2: PERSONAL HEALTH

2.1 Do you currently, or have you taken any antibiotics in the past 3 months?
YES NO CURRENT

If so, can you recall the name? ___________________________

2.2. Do you smoke? YES NO

If yes, what is the average number of cigarettes you smoke each day?

____

2.3 Do you regularly take a probiotic – these are most commonly dairy products such as Yakult or Kefir drinks, or Yogurts like Activia that are described as having 'active cultures' and are often taken to improve digestion or health ? YES NO

If yes, do you know the brand? _________________

2.4 Do you eat yogurt regularly? NO, YES

If yes, how much do you consume in an average week? _________________

SECTION 3: FEELINGS AND EMOTIONS

In this part of the interview I will be asking you about some of the emotions you may feel when you experience certain things in your life.

3.1 Please answer the following set of questions about how you view yourself.

	Disagree	Somewhat Disagree	Neither Agree nor Disagree	Somewhat Agree	Agree
**1. I feel that I have many good qualities.**	1	2	3	4	5
**2. I feel that I am a failure.**	1	2	3	4	5
**3. I have a positive attitude about myself.**	1	2	3	4	5
**4. I feel worthless.**	1	2	3	4	5
**5. I am satisfied with myself.**	1	2	3	4	5
**6. I think I am no good at all.**	1	2	3	4	5

3.2 How much do you agree or disagree with each of the following statements?

	Disagree	Somewhat Disagree	Somewhat Agree	Agree
**1. I get uncomfortable when someone wants to be very close.**	1	2	3	4
**2. I often worry that my partner doesn't really love me.**	1	2	3	4
**3. I have trouble getting others to be as close as I want them to be.**	1	2	3	4
**4. When I am close to someone it gives me a sense of comfort about life in general.**	1	2	3	4
**5. I find it easy to be close to others.**	1	2	3	4
**6. I often worry my partner will not want to**	1	2	3	4

stay with me.				
7. I find others do not want to get as close as I would like.	1	2	3	4
8. It feels relaxing and good to be close to someone.	1	2	3	4
9. I prefer not to be close to others.	1	2	3	4
10. I worry about others abandoning me.	1	2	3	4
11. My desire to get very close sometimes scares people away.	1	2	3	4
12. Being close to someone gives me a source of strength for other activities.	1	2	3	4
13. I am very comfortable being close to others.	1	2	3	4
14. Others want me to get closer than I feel comfortable being.	1	2	3	4

3.3 Please answer the following set of questions about the people in your life that you depend on.

	Disagree	Somewhat Disagree	Neither Agree nor Disagree	Somewhat Agree	Agree
1. There is a special person who is around when I am in need.	1	2	3	4	5
2. I have a special person who is a real source of comfort to me.	1	2	3	4	5
3. My family really tries to help me.	1	2	3	4	5
4. I can talk about my problems with my family.	1	2	3	4	5
5. I have friends with whom I can share my joys and sorrows.	1	2	3	4	5
6. I can talk about my problems with my friends.	1	2	3	4	5
7. If someone wants to fight me, my family will stand by me.	1	2	3	4	5
8. There is someone I can borrow money from in an emergency.	1	2	3	4	5

**9. I can count on my partner for emotional support.**	1	2	3	4	5
**10. My partner really understands me.**	1	2	3	4	5

3.4 I am now going to ask you some questions about your point of view of different topics.

	Disagree	Somewhat Disagree	Neither Agree nor Disagree	Somewhat Agree	Agree
**1. I believe an understanding of the past is essential for everyday life.**	1	2	3	4	5
**2. I believe I could do just about anything I really set my mind to.**	1	2	3	4	5
**3. I believe that if you don't know about your people's past it is hard to figure out where you belong.**	1	2	3	4	5
**4. I believe that if you understand your connection to the earth you will experience balance and harmony in your life.**	1	2	3	4	5
**5. I believe that if you just let things happen everything will work out for the best.**	1	2	3	4	5
**6. I believe that when I make plans, I am almost certain I can make them work.**	1	2	3	4	5
**7. Personal wealth is much less important than spiritual growth.**	1	2	3	4	5
**8. I am usually able to protect my personal interests.**	1	2	3	4	5
**9. If you live a good life, good things will happen to you.**	1	2	3	4	5
**10. If you are a spiritually powerful person, you will have good fortune.**	1	2	3	4	5

**11. It is important to accept life as it comes.**	1	2	3	4	5
**12. When I get what I want it is usually because I worked hard for it.**	1	2	3	4	5
**13. Even if it seems like a hardship, everything in life happens for a reason.**	1	2	3	4	5

3.5 Below is a list of losses that you and your family may have experienced. Other American Indians may have also experienced these losses. Please indicate how frequently you think about each of these losses.

	Never	Yearly at special times	Monthly	Weekly	Daily	Several times a day
**1. Loss of our land**	1	2	3	4	5	6
**2. Loss of our language**	1	2	3	4	5	6
**3. Loss of our traditional spiritual ways**	1	2	3	4	5	6
**4. Loss of our family ties because of boarding school or going away to school**	1	2	3	4	5	6
**5. Loss of family from their lands to government relocation or policies**	1	2	3	4	5	6
**6. Loss of respect from poor treatment by government officials**	1	2	3	4	5	6
**7. Loss of trust in whites from broken treaties**	1	2	3	4	5	6

**8. Loss of our culture**	1	2	3	4	5	6
**9. Losses from the effects of alcoholism on our people**	1	2	3	4	5	6
**10. Loss of respect by children and grandchildren for elders**	1	2	3	4	5	6
**11. Loss of our people through early death**	1	2	3	4	5	6
**12. Loss of respect by children for traditional ways**	1	2	3	4	5	6

3.6 Now we would like to ask you how you feel when you think about these losses. How frequently do you have these feelings about these losses?

	Never	Seldom	Sometimes	Often	Always
**1. Often feel sadness or depression**	1	2	3	4	5
**2. Often feel anger**	1	2	3	4	5
**3. Often feel anxiety or nervousness**	1	2	3	4	5
**4. Uncomfortable around white people when you think of these losses**	1	2	3	4	5
**5. Shame when you think of these losses**	1	2	3	4	5
**6. Loss of concentration**	1	2	3	4	5
**7. Feel isolated or distant from other people when you think of these losses**	1	2	3	4	5
**8. A loss of sleep**	1	2	3	4	5
**9. Rage**	1	2	3	4	5

**10. Fearful or distrustful of the intentions of white people**	1	2	3	4	5
**11. Feel like it is happening again**	1	2	3	4	5
**12. Feel like avoiding places or people that remind you of these losses**	1	2	3	4	5

3.7 **Now think about the past seven days. How often has each of the following things been true <u>during the past seven days?</u>**

	Never or rarely	Sometimes	A lot of the time	Most of the time or all of the time
**1. You were bothered by things that usually don't bother you.**	1	2	3	4
**2. You could not shake off the blues, even with help from your family and your friends, during the past seven days.**	1	2	3	4
**3. You felt that you were just as good as other people, during the past seven days.**	1	2	3	4
**4. You had trouble keeping your mind on what you were doing, during the past seven days.**	1	2	3	4
**5. You were depressed, during the past seven days.**	1	2	3	4
**6. You were too tired to do things, during the past seven days.**	1	2	3	4
**7. You enjoyed life, during the past seven days.**	1	2	3	4
**8. You were sad, during the past seven days.**	1	2	3	4
**9. You felt that people disliked you, during the past seven days.**	1	2	3	4

__SECTION 4: LIFE EVENTS__

4.1 Life Events are the next set of questions. For each life event, answer “yes” if the event happened to you in the past year, or “no” if the life event did not happen to you in the *__past year__*.

	No	Yes
**1. Has a family member called you names, insulted you, or shamed you on a regular basis?**	0	1
**2. Was your partner very jealous of you a lot of the time?**	0	1
**3. Was your partner stingy toward you a lot of the time?**	0	1
**4. Have you lived with someone who spent a lot of money gambling?**	0	1
**5. Has a member of your partner’s family called you names, insulted you, or shamed you on a regular basis?**	0	1
**6. Have you wanted to work but could not find a job or lost a job?**	0	1
**7. Has your partner wanted to work but could not find a job or lost a job?**	0	1
**8. Have you worried about getting kicked out of school, or what your parents would say about your school performance?**	0	1
**9. Have you worried about doing poorly in sports?**	0	1
**10. Were you or a close family member threatened or hurt by gang violence?**	0	1
**11. Have people gossiped or spread rumors about you?**	0	1
**12. Has someone important to you attempted suicide and survived?**	0	1
**13. Has someone important to you committed suicide?**	0	1
**14. Have you had a serious argument with a friend or friends?**	0	1
**15. Have you broken up with your partner?**	0	1
**16. Have you gotten in a car wreck in which someone was hurt or killed?**	0	1
**17. Has a relative taken over the care of your child (or children)?**	0	1
**18. Have you lost custody of your child (or children)?**	0	1
**19. Were you unable to find good child care for your child (or children)?**	0	1
**20. Has someone important to you died from causes other than suicide?**	0	1
**21. Have you or someone important to you had an alcohol or drug problem?**	0	1
**22. Have you or someone important to you gone to an alcohol or drug treatment program?**	0	1
**23. Have you or someone important to you “fallen off the wagon”?**	0	1
**24. Have you or someone important to you had a serious health problem or injury or was hospitalized (not including alcohol or drug problems or treatment)?**	0	1

4.2 Please answer "yes" if any of the following have happen to you.

	No	Yes
**1. Have you ever attempted suicide?**	0	1
**2. Did you attempt suicide in the past year?**	0	1
**3. Have you ever seriously considered committing suicide?**	0	1
**4. Did you seriously consider committing suicide in the past year?**	0	1

SECTION 5: SEXUAL BEHAVIORS, CONTRACEPTION AND RISK FACTORS

We are now almost finished with the interview. I would like to ask you some questions about your personal hygiene and sex life. We know that some of these questions may be sensitive. We want to remind you that your answers are confidential and anonymous. Nothing you tell us will be associated with your name.

5.1 Are you currently sexually active with a partner? YES NO

If yes what kind of relationship is it?

Husband

Boyfriend/girlfriend
Friend/Some one you know
A Hook- Up
Authority figure (teacher, boss, etc.)
Family member/relative
Stranger

Other (please specify) ________________________________

If yes, how long have you been with your current sex partner? __________

5.2 Do you douche? YES NO
If yes, how many days has it been since you last douched? ______________

5.3 Have you had a urinary tract infection in the past 3 months? YES NO
If yes, approximately how many days ago? ______________

5.4 To your knowledge have you ever been diagnosed with Bacterial Vaginosis? YES NO
If yes when? _______________________

5.5 When was your last menstrual period? _______________

5.6 Is your menstruation regular? YES NO

5.7 What is the approximate length of your menstrual cycle (number of days)? _______________

5.8 Are you currently pregnant? YES NO
If yes how many pregnancies have you had? _______

If >0, how many children have you had? __________ ______________

5.9 Have you had a hysterectomy? YES NO

5.10 Have you experienced any of the following in the past 6 weeks?

Vaginal discharge	YES	NO
Vaginal odor	YES	NO
Vaginal pain	YES	NO
Vaginal itching	YES	NO

If yes, how many days ago, approximately? _______________

5.11 What gender do you usually have sex with?

Men only or primarily

Women only or primarily

Both more or less equally

5.12 During your <u>lifetime</u>, how many people have you had sex with? _______ [# of people]

5.13 During the <u>past month</u> how many people have you had sex with? _____ [# of people]

5.14 What is the average number of times you have had sex (vaginal, oral or anal) in the past month? _____________

5.15 The last time you had sex did you or the person you had sex with use alcohol and/or drugs when you had sex?

0. No.
1. Yes. If yes, please explain. Which one of you used alcohol/drugs and what alcohol and/or drugs were used?

____

5.16 What kind of birth control do you currently use? *CIRCLE ALL THAT APPLY.*

1. the birth control pill
2. a condom
3. spermicide (cream, foam, or suppositories)
4. diaphragm, cervical cap, contraceptive sponge

5. Depo-Provera (Depo shot) or Noristerat injection, Norplant implant, IUD

6. rhythm method (or periodic abstinence/not having sexual intercourse during fertile times)

7. sterilization (such as tubal ligation or sex partner's vasectomy)

8. withdrawal (pulling the penis out before ejaculation)

9. other (Please write in.)

____

10. We did not use a birth control method the last time we had sexual intercourse.

5.17 The last time you had sex what kind of birth control did you use? ____________

5.18 During the <u>past year</u>, have you been tested for an STI? YES NO

If yes, did you have an STI? YES NO

If yes, what kind of STI was it? ____________________

If yes, have you had an STI before this one? YES NO

If yes, how many times have you had an STI before this one? ____________

5.21 Have you ever tested positive for HIV? YES NO

5.22 During the <u>past year</u>, have you had sex with a man/woman who:

	YES	NO
**1. You knew or suspected was having sex with another man/woman?**	1	0
**2. You knew or suspected shared needles to shoot drugs?**	1	0

**3. You knew or suspected had an STI?**	1	0
**4. You knew or suspected had HIV or AIDS?**	1	0
**5. You knew or suspected had Hepatitis C?**	1	0

5.23 If you needed medical help with anything related to your reproductive health such as if you needed birth control or treatment for an STI where would you go? And why would you go there?

Probe: If participant would go to IHS? Why or why not?

SECTION 6: CLOSING STATEMENT

There is one final question we would like to ask you related to participating in this study. We understand that we have asked you several sensitive and very private questions about yourself and we have also asked you to provide us with vaginal samples, which we also understand is very personal.

6.1 Could you please tell us what it was like for you to participate in this study? For example was it easy or difficult? And tell us why you felt this way?

That was your last question. Thank you so much for helping us out. The information you gave us is very important to help us better understand how emotions, exposure to stressful events in your life and life experiences influence microbiota and hormones. As I said in the beginning, everything you told me today will remain private. Your name will not be on any of the information. Please make sure we have your contact information so we can get in touch with you when we have the results of your vaginal sample test and interview responses.
